# Characterizing SV40-hTERT Immortalized Human Lung Microvascular Endothelial Cells as Model System for Mechanical Stretch-Induced Lung Injury

**DOI:** 10.3390/ijms26020683

**Published:** 2025-01-15

**Authors:** Beatrix Hochreiter, Claudia Lindner, Matthias Postl, Eva Hunyadi-Gulyas, Zsuzsanna Darula, Oliver Domenig, Smriti Sharma, Irene M. Lang, Attila Kiss, Andreas Spittler, Konrad Hoetzenecker, Roman Reindl-Schwaighofer, Katharina Krenn, Roman Ullrich, Matthias Wieser, Regina Grillari-Voglauer, Verena Tretter

**Affiliations:** 1 Clinical Division of General Anaesthesia and Intensive Care Medicine, Department of Anesthesia, Genera Intensive Care and Pain Therapy, Medical University Vienna, 1090 Vienna, Austria; beatrix.hochreiter@meduniwien.ac.at (B.H.); roman.ullrich@auva.at (R.U.); 2 Evercyte GmbH, 1110 Vienna, Austria; claudia.lindner@phoenestra.com (C.L.); matthias.postl@evercyte.com (M.P.); matthias.wieser@evercyte.com (M.W.); regina.grillari@evercyte.com (R.G.-V.); 3 Core Facility Proteomics Research Group, HUN-REN Biological Research Centre, 6726 Szeged, Hungary; gulyas.eva@brc.hu (E.H.-G.); zsuzsanna.darula@hcemm.eu (Z.D.); 4 Single Cell Omics Advanced Core Facility, Hungarian Centre of Excellence for Molecular Medicine, 6728 Szeged, Hungary; 5 Attoquant Diagnostics, 1110 Vienna, Austria; oliver.domenig@attoquant.com; 6 Division of Cardiology, Department of Internal Medicine II, Vienna General Hospital, Medical University Vienna, 1090 Vienna, Austria; smriti.sharma@meduniwien.ac.at (S.S.); irene.lang@meduniwien.ac.at (I.M.L.); 7 Center for Biomedical Research and Translational Surgery, Medical University Vienna, 1090 Vienna, Austria; attila.kiss@meduniwien.ac.at; 8 Core Facility Flow Cytometry and Department of Surgery, Medical University Vienna, 1090 Vienna, Austria; andreas.spittler@meduniwien.ac.at; 9 Department of Thoracic Surgery, Medical University of Vienna, 1090 Vienna, Austria; konrad.hoetzenecker@vumc.org; 10 Clinical Division of Nephrology and Dialysis, Department of Internal Medicine III, Medical University Vienna, 1090 Vienna, Austria; roman.reindl-schwaighofer@meduniwien.ac.at

**Keywords:** mechanical ventilation, mechanical stretch, ventilator-induced lung injury, renin–angiotensin system, pulmonary endothelium, microvascular endothelial cell line

## Abstract

Drug development for human disease relies on preclinical model systems such as human cell cultures and animal experiments before therapeutic treatments can ultimately be tested on humans in clinical studies. We here describe the generation of a novel human cell line (HLMVEC/SVTERT289) that we generated by transfection of microvascular endothelial cells from healthy donor lung tissue with the catalytic domain of telomerase and the SV40 large T/small t-antigen. These cells exhibited satisfactory growth characteristics and largely maintained their native characteristics, including morphology, cell surface marker expression, angiogenic potential and the protein composition of secreted extracellular vesicles. In order to test their suitability as a disease model, we simulated mechanical stress induced by cyclic stretch as encountered in ventilator-induced lung injury using the FlexCell^®^ system and compared their performance to primary lung endothelial cells. In this setting, HLMVEC/SVTERT289 cells exhibited significantly higher neprilysin activity on the cell surface and extracellular vesicles secreted from the cell line exhibited higher Tissue Factor and ACE2 expression but lower ACE expression and ACE activity than vesicles released from the primary cells. This study provides an unprecedented and detailed characterization of the HLMVEC/SVTERT289 cell line, which should help to appraise its suitability in different molecular studies.

## 1. Introduction

The human lung displays a large number of different cell types, which recently have been addressed by different single cell analysis approaches and have been mapped in integrated cell atlases [1,2]. An important feature is the exceptionally high vascular density, which facilitates proper gas exchange. The gas-exchange surface is made up of thin regions, where the alveolar epithelium (containing alveolar type I and type II cells) is tightly attached to the endothelium cell layer, only separated by the epithelial basal membrane. Alveolar type I cells are largely expanded on the alveolar surface and are the key players in barrier function and gas exchange. They are incapable of cell division and are regenerated by the differentiation of alveolar type II cells, which also produce surfactant proteins and play an important role in host defense mechanisms and wound healing [3]. The understanding of the molecular mechanisms involved in the differentiation of ATII cells into ATI cells is still not complete, but is important to advance therapeutic options in the treatment of lung conditions with epithelial damage, such as in acute respiratory distress syndrome (ARDS) [4].

The inner vascular lining is maintained by a single layer of specialized endothelial cells forming a barrier between blood and lung tissue and fulfilling crucial functions with regard to gas exchange, vascular tone, coagulation and inflammation. These functions are executed by two intermingled endothelial cell types, aerocytes and general capillary endothelial cells, as found in a recent study by using single-cell sequencing [5]. Most pathological states of the lung involve the endothelium and can be triggered by bacterial, viral or mycotic infections, aspiration, inflammation, or oxidative or mechanical stress. Despite their unique location, pulmonary endothelial cells are still intrinsically heterogenous and fulfill different functional tasks [6]. Therefore, lung endothelial cell cultures are important model systems to study pulmonary vascular biology and its role in pathological conditions.

A number of mechanistic or pharmacological studies use primary cells or derived cell lines to understand pathological processes or test therapeutical interventions. The most representative cell culture assays try to use primary cells freshly isolated from tissue biopsies; however, human cells are especially difficult to obtain and have a very limited culture span due to early de-differentiation and replicative senescence when being cultivated in vitro. Early alternatives included lung-cancer-cell derived cell lines, which reflect some of the properties of native lung cells; however, in most cases, tumor cells undergo metabolic reprogramming and change their protein expression to support rapid proliferation, neovascularization and other untypical processes. The limited proliferation of somatic cells in vitro (maximal population doublings: Hayflick limit) varies between cell type, donor age and culture conditions and has been shown to primarily depend on an increasing lack of telomerase activity leading to telomere shortening [7,8]. One way out of this dilemma is the use of embryonic or adult stem cells, which have the capacity to self-renew and can be differentiated into specialized cell types. Restricted availability and ethical considerations, especially with regard to embryonic stem cells, and the observation that adult stem cells are frequently also limited by insufficient telomerase activity led to the successful attempt to extend the life span of primary cells by introducing telomerase reverse transcriptase (hTERT) into somatic cells [9]. While in some cases hTERT transfection alone is sufficient to bypass replicative senescence, in other cases additional expression of other genetic elements (like SV40 T antigen or HPV16 E6/E7) seems to be needed for immortalization [10,11].

We here describe the establishment of a continuously growing cell line of human pulmonary microvascular endothelial cells (named HLMVEC/SVTERT289) and present a detailed basic characterization of them, including the expression of cell surface markers and assays testing for typical endothelial properties, such as tube formation in vitro. In a novel approach, we used the cells to compare their performance with the original primary cells in a model system for mechanical stretch-induced lung injury, such as that observed during mechanical ventilation. We analyzed changes in cellular gene expression as well as the quantity and qualitative components of the extracellular vesicles released from the cells upon cyclic mechanical stress. Bioinformatic analyses were used to deduce functional implications. Extracellular vesicle research has gained significant importance in recent years, with a view towards their role as means of cell-to-cell communication, as biomarkers and their potential use in concepts of therapy. Suitable cell lines, i.e., those that keep many features of primary cells and are well characterized, facilitate mechanistical lung research, as they can be used as readily available workhorses prior to the use of primary cells or tissues.

## 2. Results

The following sections of presented results comprise the basic characterization of the HLMVEC/SVTERT289 cell line, including morphology, the presence of telomerase activity and SV40 large T expression, cell surface marker expression and the capability of tube formation, followed by the performance of the cell line in an in vitro model of ventilator-induced lung injury (VILI) simulated by cyclic mechanical stretch. The cell responses under this type of stress are compared between the cell line and primary cells. Analyses include changes in cellular gene expression, quantification and qualitative analysis of extracellular vesicles (EVs) and, finally, a detailed characterization of the enzymes of the renin–angiotensin system (RAS) and their activity on the cell surface as well as components of EVs, as the lung endothelium is a region of central importance in the functionality of the RAS.

### 2.1. Basic Characterization of the HLMVEC/SVTERT289 Cell Line

#### 2.1.1. HLMVEC and HLMVEC/SVTERT289 Cell Morphology and Transgene Expression

**Figure 1:**
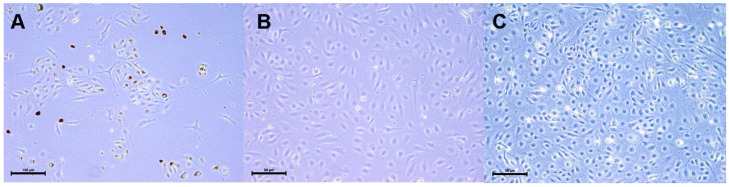
(**A**) HLMVECs isolated from human lung biopsies by magnetic separation on Dynabeads coupled to CD31 and UEA-1 lectin. Scale bar = 100 μm (**B**) hTERT- and SV40 T-Antigen-overexpressing cells (HLMVEC/SVTERT289) at passage 23. Scale bar = 50 μm. (**C**) at passage 36. Scale bar = 50 μm.

As shown in Figure 1A, the primary cell cultures from human lung tissues purified from human lung biopsies by magnetic selection using CD31 antigen and UEA-1 lectin binding represent a mixture of cells with typical endothelial morphology as well as spindle-shaped mesenchymal cells. Figure 1B,C show pure preparations of hTERT- and SV40 T-antigen-overexpressing HLMVECs at passages 23 and 36, respectively. Primary endothelial cells and cells overexpressing the immortalizing genes show a very similar morphology, demonstrating that the immortalization process does not alter cellular morphology. Lifespan-extended HLMVEC/SVTERT289 cells maintain a phenotype similar to the corresponding primary cells.

**Figure 2:**
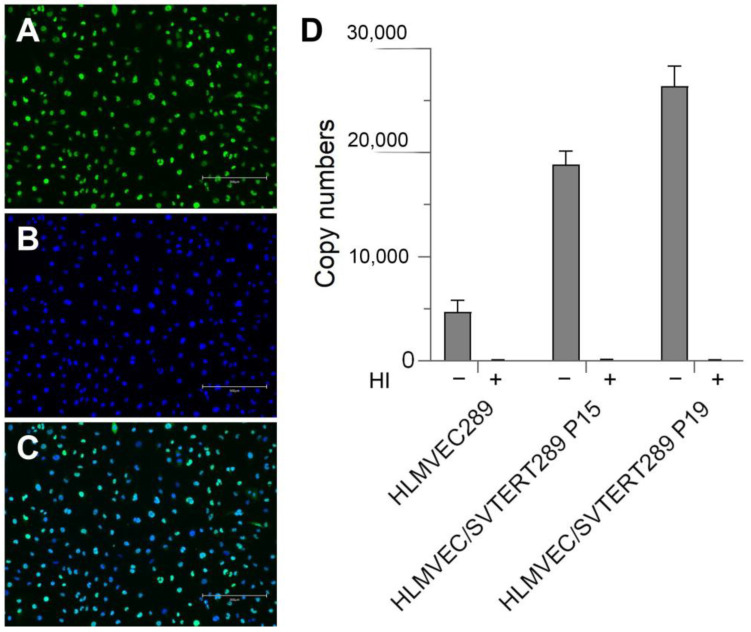
Transgene expression in HLMVEC/SVTERT289 cells: Indirect immunofluorescence demonstrates homogenous expression of SV40 large T. (**A**) SV40 large T (AF488, green) is detected in the nucleus. (**B**) DNA counterstain (Vybrant dye, blue). (**C**) Merge picture. Scale bar = 300 μm. (**D**) Telomerase activity of non-telomerized HLMVEC289 and telomerized HLMVEC/SVTERT289 cells (TRAP assay). Heat inactivation (HI = heat-inactivated, right bar) abolished telomerase activity completely. TERT-transfected cells showed a clear induction of telomerase activity. Error bars indicate the standard deviation. The y-axis displays copy numbers of the telomeric repeats of each sample, calculated according to manufacturer’s protocol.

Whereas normal cells can only be propagated for 3–12 population doublings (PDs) before entering replicative senescence, cells overexpressing the SV40 early region and hTERT are grown for over 35 PDs. The transfection of HLMVECs resulted in homogeneous expression of SV40 large T, as demonstrated by immunofluorescence in HLMVEC/SVTERT289 cells at P15 (Figure 2A–C). After transfection, the cells also showed robust induction of telomerase activity (Figure 2D). Analysis of the cell-specific telomerase activity of normal HLMVEC289 cells (from the same donor) and telomerized HLMVEC/SVTERT289 cells at different passages was performed via a TRAP assay. Transfected endothelial cells displayed a clear induction of telomerase activity when compared to the normal (non-transfected) endothelial cells (HLMVEC289).

#### 2.1.2. Microvascular Endothelial Marker Expression

**Figure 3:**
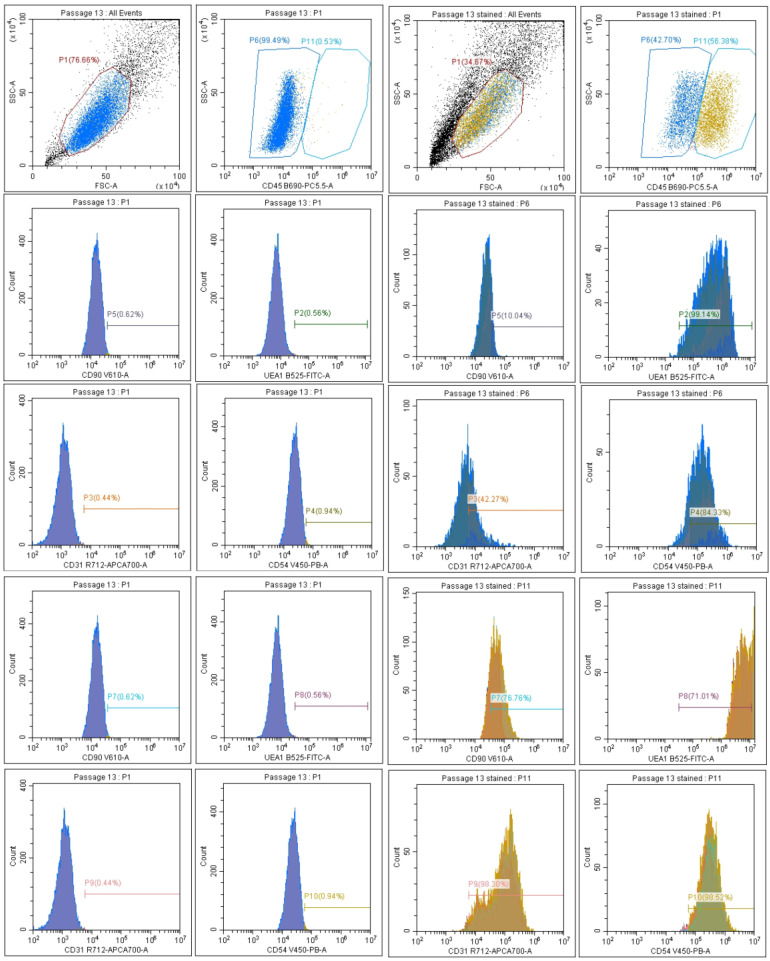
Characterization of the surface antigens CD31, CD45, CD54, CD90 and UEA-1 of HLMVEC/SVTERT289 cells by flow cytometry (passage 20). Cells from individual gates are shown in different colors. (Fraction of UEA1-positive cells is underestimated in the yellow gate, as these cells are very strongly positive for this epitope, exceeding the scale of the x-axis).

HLMVEC/SVTERT289 cells at passage 20 were analyzed for the expression of surface antigens CD31, CD45, CD54 and UEA-1 by flow cytometry (Figure 3).

#### 2.1.3. Tube Formation of HLMVEC/SVTERT289 Cells

**Figure 4:**
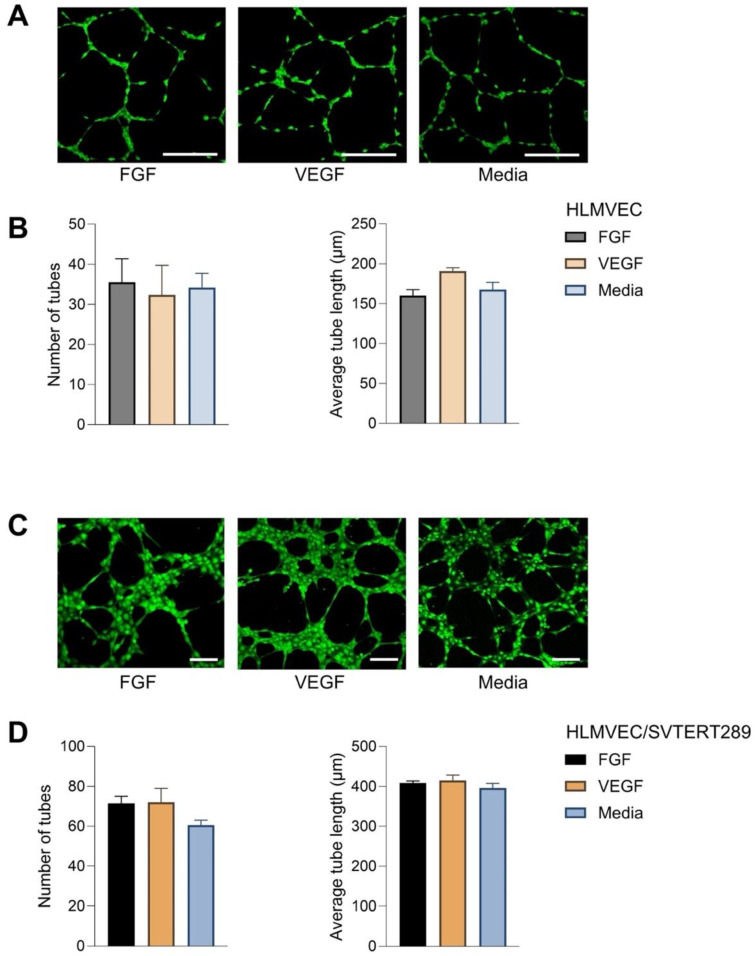
In vitro tube formation assay of HLMVECs and HLMVEC/SVTERT289 cells. Representative images (**A**) and quantification (**B**) of tube formation in primary HLMVECs stimulated with bFGF (50 ng/mL) and VEGF (50 ng/mL) or without stimulation (media) for 16 h. Representative images (**C**) and quantification (**D**) of tube formation in the HLMVEC/SVTERT289 cell line stimulated with bFGF (50 ng/mL) and VEGF (50 ng/mL) or without stimulation (media) for 4 h. The graphs in (**B**,**D**) represent mean values and error bars indicate the standard deviation. Scale bars: (**A**) 100 μm and (**C**) 200 μm.

The tube formation assay is an in vitro assay for angiogenesis, a typical capability of endothelial cells and used here to prove that the immortalized cell line has retained this function. Angiogenic signals such as basic fibroblast growth factor (bFGF) or vascular endothelial growth factor-A (VEGF-A) induce the formation of tubes when endothelial cells are plated on a basement membrane matrix. Quantification is possible by measuring the number of branch sites and nodes, loops and meshes or the length of the tubes. Like primary HLMVECs, HLMVEC/SVTERT289 cells possess angiogenic potential when plated on the basement membrane matrix and can be stimulated by VEGF and bFGF to form neoangiogenic networks (Figure 4).

### 2.2. Cyclic Mechanical Stretch as In Vitro Model for VILI

#### 2.2.1. Analysis of Cellular Gene Expression

**Figure 5:**
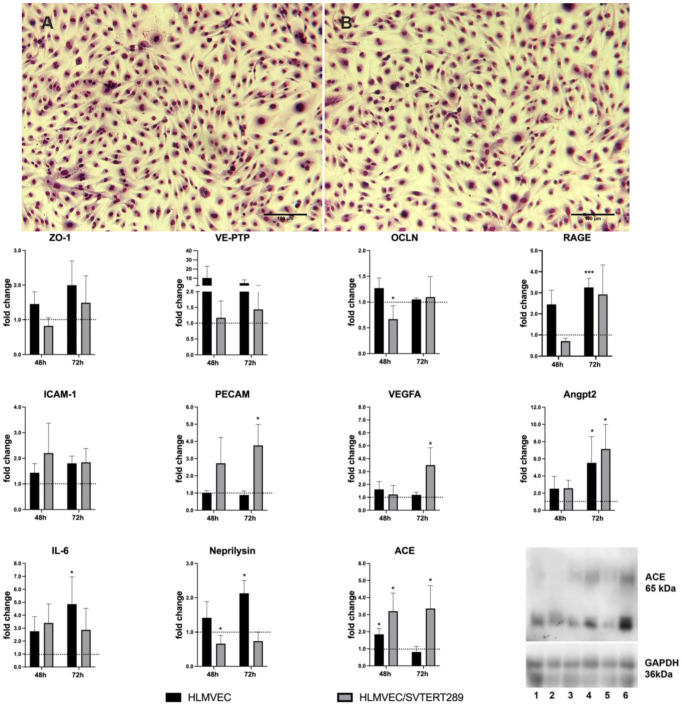
Exposure to cyclic stretch by FlexCell^®^. HLMVECs and HLMVEC/SVTERT289 cells were exposed to cyclic stretch with 21% extension for up to 72 h. The top panels show microscopic images of HLMVEC/SVTERT289 cells visualized with hemacolor^®^ rapid stain (**A**) without stretch and (**B**) after 24 h sinus-curve-like cyclic stretch. Scale bar = 100 μm. The number of cells remaining on the membrane decreased with prolonged exposure. Bar charts show RNA gene expression analysis in primary HLMVEC and HLMVEC/SVTERT289 cell lysates after 48 h and 72 h of cyclic stretch, as quantified by qRT-PCR. Graphs show “fold-change” (mean values; error bars indicating the standard deviation) of mRNA expression relative to house-keeping gene ß-actin and control condition. The mean value of the control condition (no stretch) is represented by the dotted line at fold change = 1.0, which separates downregulated expression (fold change < 1.0) from upregulated expression (fold change > 1.0) under cyclic stretch. For the control condition, cells were plated from the same batch and at the same density but were left in the incubator without stretching. Data were obtained from 3 independent experiments. (Gene symbols are explained under “Abbreviations”) * *p* < 0.05; *** *p* < 0.001 (one-sample *t*-test). The bottom right panel shows Western blot analysis of the ACE protein (low molecular isoform) and GAPDH as the loading control. Treatments were as follows: (1) unstretched 24 h; (2) stretched for 24 h; (3) unstretched for 48 h; (4) stretched for 48 h; (5) unstretched for 72 h; and (6) stretched for 72 h.

Cells were plated on collagen I-coated cell culture plates suitable for cyclic mechanical stretch in a Flexcell^®^ apparatus. We investigated changes in gene expression in cell lysates after 48 h and 72 h of sinus-curve-like stretching (Figure 5), as well as selected surface exposed antigens on secreted vesicles by flow cytometry and the total protein content of extracellular vesicles by quantitative proteomic analysis.

#### 2.2.2. Release of Extracellular Vesicles: Proteomic Analysis of EVs

It is generally believed, that all cell types release EVs of different origin and size (exosomes, microvesicles and apoptotic bodies) that are loaded with specific cargo depending on the (stress) condition of the mother cells. EVs are mediators of downstream biological functions and can have harmful or beneficial effects. Embedded in their lipid membrane, EVs carry surface antigens of their mother cells; therefore, it is feasible to identify their origin in the circulation. EVs released from the pulmonary endothelium have been shown to play an important role in pathological lung conditions such as ARDS, pulmonary hypertension (PH), chronic obstructive pulmonary disease (COPD) and obstructive sleep apnea syndrome (OSAS).

**Figure 6:**
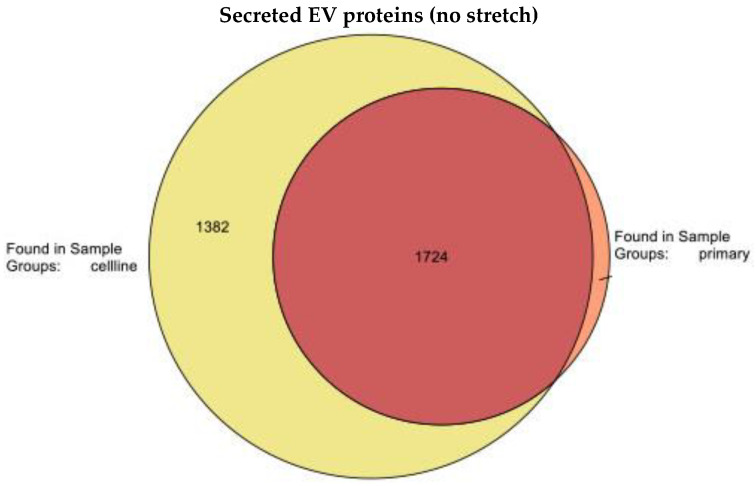
Visualization of identified proteins and their intersection in EVs from HLMVECs (red) and HLMVEC/SVTERT289 cells (yellow) after 48 h culture without cyclic extension.

In order to analyze the protein content of EVs from both cell types, we intended to analyze the full proteome of EVs released from primary HLMVECs and HLMVEC/SVTERT289 cells under standard culture conditions in serum-free medium (48 h, incubator, 37 °C; 5% CO_2_). Proteomic analysis resulted in the identification of 1783 proteins in the primary HLMVECs and 3106 proteins in the HLMVEC/SVTERT289 cells with an overlap of 1724 proteins identified in both cell types (Figure 6).

**Figure 7:**
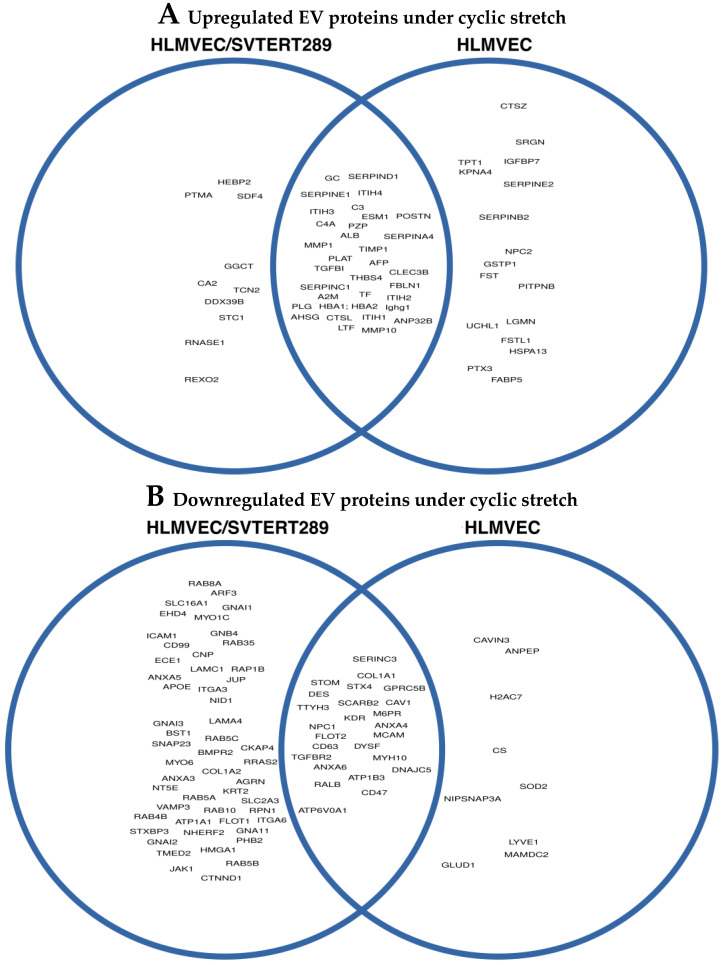
Visualization of significantly (**A**) upregulated proteins or (**B**) downregulated proteins in extracellular vesicles released from HLMVEC/SVTERT289 cells and HLMVECs, respectively, upon 48 h exposure to cyclic stretch (relative to control condition = no stretch). Venn diagrams show overlap of significantly changed proteins in both cell types.

We further seeded both cell types at the same density and exposed them to the same regimen of cyclic stretching (48 h 21% extension). After 48 h, we isolated supernatants (6 mL each) and concentrated them to a 500 μL final volume. A BCA protein test gave the following mean protein concentrations from 3 replicates: primary HLMVECs: 658 ± 86 μg/mL; HLMVEC/SVTERT289 cells: 891 ± 12 μg/mL, implicating 35% more protein in the latter. The proteins significantly upregulated in EVs after 48 h cyclic stretch are shown in Figure 7A and the proteins significantly downregulated are shown in Figure 7B with overlapping proteins visualized in Venn diagrams.

**Figure 8:**
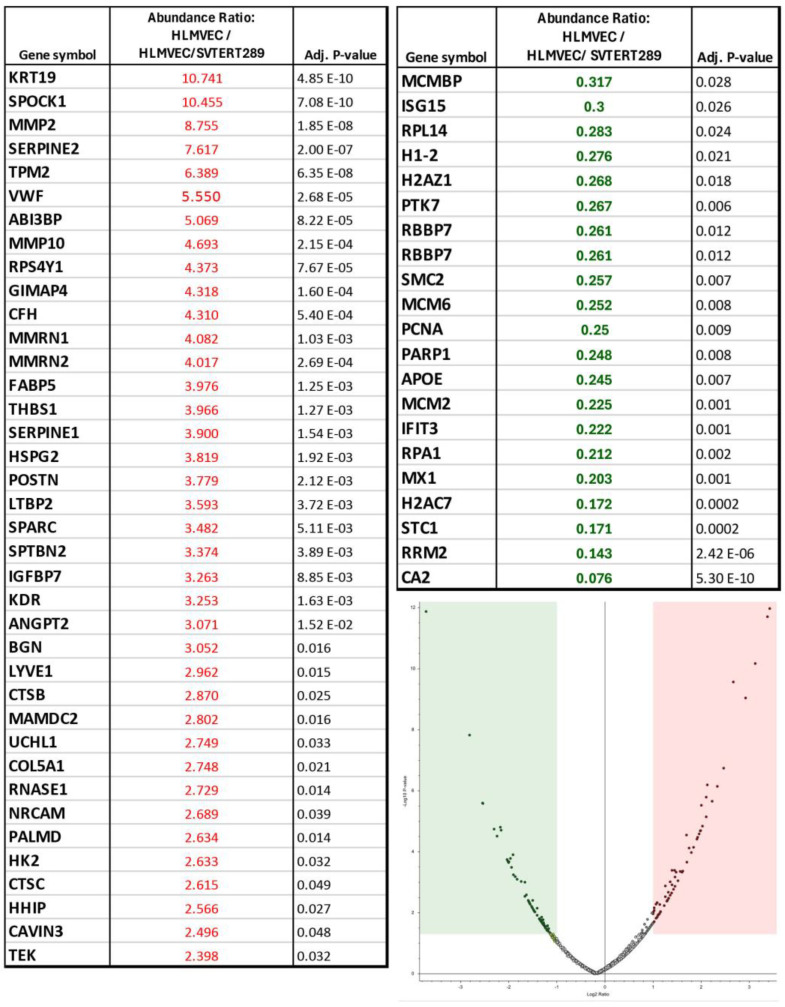
Comparison of protein expression in EVs from HLMVECs and HLMVEC/SVTERT289 cells. Overrepresented proteins in EVs from HLMVECs compared to HLMVEC/SVTERT289 cells are shown in red and underrepresented proteins are shown in green (*p* < 0.05).

The quantitative differences of EV proteins from HLMVECs relative to those from HLMVEC/SVTERT289 cells after 48 h of 21% extension are shown in the volcano plot of Figure 8 and the included table. Proteins with higher abundance in EVs from HLMVECs are highlighted in red and proteins with lower abundance in EVs from HLMVECs are highlighted in green (*p* < 0.05).

In order to deduce the functional implications of proteins that are significantly up- or downregulated in EVs under cyclic stretch relative to the control condition (incubation with no stretch), we performed enrichment analyses using the Reactome database and the significantly up- or downregulated proteins as protein entries (Supplementary Data S1; Tables S1–S4).

Significant functional terms overlapped to quite a large extent, especially for the top hits between EVs from HLMVECs and HLMVEC/SVTERT289 cells. The upregulated proteins are implicated in hemostasis, the clotting cascade and platelet function, extracellular matrix degradation, complement activation, lipoproteins, lipid and vitamin D metabolism and O_2_/CO_2_ exchange at erythrocytes.

Downregulated proteins are likewise implicated in hemostasis and platelet function, but also in VEGF signaling, eNOS function and trafficking and interaction with the neuronal system (GABA and GABA-receptors). Downregulated proteins in EVs from primary HLMVECs were uniquely involved in the conversion of angiotensinogen to angiotensin metabolites, TGF-ß receptor signaling (involved in EMT) and p38 MAPK signaling. Downregulated proteins in EVs from HLMVEC/SVTERT289 cells were uniquely implicated in integrin cell surface interactions, G-protein and adenylate cyclase activation, vesicle budding (both from the trans-Golgi network and clathrin-mediated budding), as well as cell junctions (adherens junctions) and Thromboxane signaling.

#### 2.2.3. Release of Extracellular Vesicles: Flow Cytometry Analysis of EV Surface Proteins

**Figure 9:**
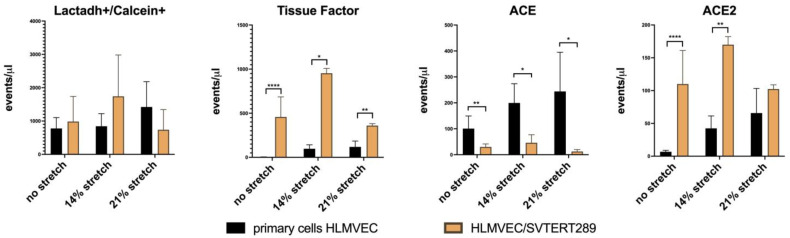
Flow cytometry analysis of EVs released from primary HLMVECs and HLMVEC/SVTERT289 cells under basal culture conditions or under cyclic stretch for 48 h with 14% or 21% extension, respectively *(n* = 3 experiments). Bars indicate mean values; error bars indicate standard deviation. * *p* < 0.05; ** *p* < 0.01; **** *p* < 0.0001 (Student’s *t*-test).

We used flow cytometry to quantify phosphatidyl-serine-positive EVs (=total number of microvesicles that bind Lactadherin) and to analyze the EV surface expression of Tissue Factor and the RAS components ACE and ACE2 under standard culture conditions and under cyclic stretch with two amplitudes (48 h of 14% and 21% stretch) (Figure 9).

Flow cytometry analysis for the known endothelial EV antigens Tissue Factor, ACE and ACE2 revealed differences between primary HLMVECs and immortalized HLMVEC/SVTERT289 cells. The latter show significant amounts of Tissue Factor-positive EVs under basal conditions, which is absent in the supernatants from primary cells. Also, the abundance of ACE and ACE2 in the EV-containing supernatants is reciprocal within the two cell types, with ACE predominating in EVs from primary cells and ACE2 predominating in EVs from HLMVEC/SVTERT289 cells. Cyclic stretching has some effect on the abundance of these EVs (Figure 9).

#### 2.2.4. Analysis of Components of the Renin–Angiotensin System in the VILI Model (Cell Surface and EVs)

**Figure 10:**
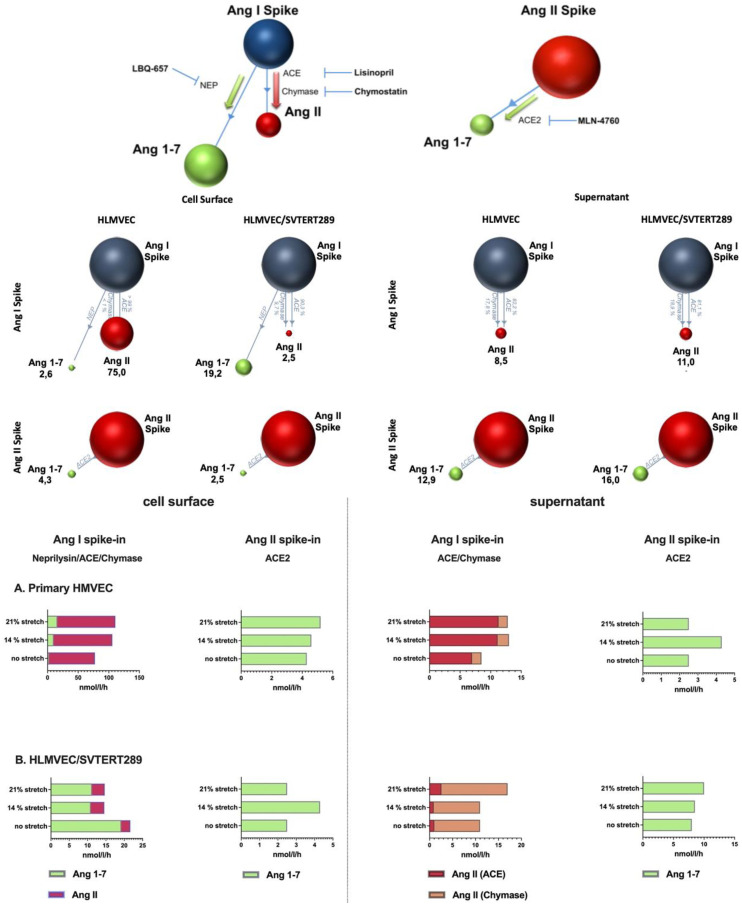
RAS enzyme activity assay on the cell surface of and on stretch-induced EVs released from HLMVECs and HLMVEC/SVTERT289 cells. The relative contributions of individual RAS enzyme activities to the formation of RAS metabolites were assessed by including specific enzyme inhibitors (see top of figure). Sphere diagrams semi-quantitatively visualize conversion of RAS metabolites, with numbers indicating metabolite formation in nmol/h when cells were not exposed to stretching. The bar charts below quantitatively show the mean values of formed metabolites from 2 and 3 individual experiments, respectively, each performed at least in technical duplicates.

The activities of the RAS-related enzymes ACE, ACE2, chymase and neprilysin were analyzed on the cell surfaces of HLMVECs and HLMVEC/SVTERT289 cells, as well as in supernatants concentrated by ultrafiltration (Figure 10). The schematic representation of the assay principle and the inhibitors used to quantify individual RAS enzyme activity is shown at the top of Figure 10.

## 3. Discussion

In this study, we describe the generation of a new human lung microvascular endothelial cell line and its thorough characterization with regard to basic endothelial properties, as well as its performance for mechanistic studies in a model of mechanical stress, as encountered in lungs of a ventilated patient.

Mechanical ventilation is frequently used at intensive care units to maintain proper blood oxygenation in patients with acute lung injury or ARDS, which can develop as a consequence of various insults, such as trauma, sepsis, pneumonia viral infection and others. The mechanical strain imposed on the lung by this intervention can be injurious by itself, leading to ventilator-induced lung injury; therefore, efforts are made in the clinic to keep it as low as possible (protective ventilation mode) [12].

The typical indicators of VILI are the release of inflammatory mediators, barrier disruption [13], activation of the renin–angiotensin-system (RAS) [14] and other forms of endothelial dysfunction such as glycocalyx shedding [15] and endothelial nitric oxide synthase uncoupling [16,17,18]. All types of cells are believed to shed vesicles under basal conditions and even more under stress such as cyclic stretch. These extracellular vesicles either bud from the outer plasma membrane of the mother cells or originate from the inner endosomal system. Extracellular vesicles in many cases mirror the status of the mother cell and therefore those released under cellular stress frequently contain cargo proteins, lipids and RNAs that induce pro-inflammatory mechanisms, affect coagulation, inhibit NO synthesis and lead to endothelial impairment [18].

Cell cultures are the first-line model systems for mechanistic studies in order to dissect pathomechanisms or to develop therapeutic interventions. Culturing a single cell type simplifies observations but also has drawbacks, as primary cells frequently show early senescence and derived cell lines have altered characteristics.

In this study, we developed a new pulmonary microvascular endothelial cell line from tissue biopsies of a healthy donor lung by transfecting isolated primary cells with plasmids containing hTERT and SV40 T/t-antigen. The feasibility of this approach has already been described by Krump-Konvalinkova et al. in 2001 [19], and the authors later provided a basic characterization of their new cell line showing superior typical endothelial properties compared to other available endothelial cell lines [20]. We here attempted to assess the suitability of our own new cell line (HLMVEC/SVTERT289) as a valid model system to study VILI in vitro using the FlexCell^®^ system. In our analyses, we compared the performance of HLMVEC/SVTERT289 cells with primary HLMVECs.

HLMVEC/SVTERT289 cells show a significantly extended life span, with an almost linear curve of population doubling. The cells maintained their cobble stone appearance beyond passage 36. Cell surface phenotyping by flow cytometry revealed some heterogeneity, with revealing endothelial characteristics (43% of the population at passage 20 were CD45 and CD90 negative, 84% were CD54 positive and 42% were CD31 positive). Another population was positive for CD45 and 77% were CD90 positive. In addition, there was over 90% CD31 and CD54 positivity. All cells exhibited UEA1 lectin staining, despite a stronger fluorescence in the CD45-positive cells. An explanation for these expression shifts might be that HLMVECs undergo some extent of endothelial–mesenchymal transition (EndMT) during culture prior to isolation and transfection, in addition to further dedifferentiation when transfected with SV40 T-antigen [21]. Expression of CD45 has been shown to be involved in EndMT processes, at least in the heart and in atherosclerosis [22,23]. CD90 is expressed by activated endothelial cells [24] and also by lung lymphatic endothelial cells [25]. Further studies could focus on individual subpopulations isolated by cell sorting in order to observe the stability of cell surface marker expression in extended culture periods. The capacity for tube formation as an indicator for angiogenesis and as a genuine property of endothelial cells has been maintained in early passages of the cell line.

Strong mechanical stretch in the FlexCell^®^ system (21% extension) resulted in upregulation of the mRNA expression of IL-6, Angpt2, RAGE, VE-PTP, ICAM1, VEGFA and ZO1 in both primary cells and the cell line, despite some differences in time and extent. ACE was especially upregulated in the cell line and less in primary cells, whereas neprilysin was solely upregulated in primary cells but not in the cell line. PECAM1 was exclusively upregulated in the cell line. The impact of mechanical stress on the lung vasculature has been investigated in many studies (for a review see [26]). The core features in endothelial signaling in response to VILI are volu- and barotrauma, shear stress, inflammation, alterations of vascular tone, activation of the RAS and barrier disruption. Cyclic stretch has been shown to result in an increased production of inflammatory cytokines such as the interleukins 1ß, 6 and 8 and to impact vessel tone via endothelial nitric oxide synthase, cycloxygenase-2 and endothelin-1, proliferation by PDGF and VEGF, cell adhesion proteins and cell junctional proteins, such as ZO-1, occludin, VE-cadherin and its regulatory phosphatase, VE-PTP. The role of the RAS in a rodent model of VILI was shown in [14], where activation of the classical and alternative RASs was shown under increasing volume ventilation with a dose-dependent increase in ACE protein expression.

Under basal culture conditions, the cell line secreted more soluble proteins and extracellular vesicles than the primary cells, but the secreted and vesicle-bound proteins qualitatively overlapped to a large extent. Mechanical forces can enhance the number of secreted vesicles and has been suggested for the enhanced production of therapeutical EVs [27].

Cyclic stretch has further been shown to result in changes in the vesicular proteome [28]. In our study, the significantly up- and downregulated proteins overlapped with regard to many proteins between primary cells and the cell line, promising the suitability to some extent of the cell line in EV research. Functional enrichment analysis of significantly changed proteins revealed that the major enriched terms between the primary cells and cell line EVs were similar and typical for the mechanical stress by overdistention. The EVs from primary cells under cyclic stretch contained larger amounts of Von-Willebrand Factor, matrix metalloproteinases, SERPINs, angiopoietin-2 and ACE than EVs from the cell line. We further characterized surface expression of Tissue Factor, ACE and ACE2 on EVs under basal conditions and under increasing cyclic stretch by using flow cytometry. Under resting conditions, EVs from primary cells did not display Tissue Factor but did display some ACE and ACE2. This is in accordance with the absence of Tissue Factor activity on healthy resting endothelial cells. However, tumor cells frequently release EVs with active Tissue factor that are capable of activating quiescent endothelial cells and can result in coagulopathies [29,30]. In our study, 14% extension increased the surface expression of Tissue Factor only on EVs from the cell line. ACE expression increased on the surface of EVs from primary cells under increasing cyclic stretch (14% and 21% extension); ACE2 increased expression on the surface of EVs from the cell line and to a lesser extent also on EVs from primary cells with increasing amplitudes of mechanical stretch, indicating some differences in the RAS components.

In order to analyze the activity of RAS components in more detail, we quantified the enzyme activity of ACE, ACE2, neprilysin and chymase on the surface of cells and EVs via mass spectrometry of quantitative metabolite formation. In this assay, the substrate Angiotensin I can be converted to Angiotensin II by ACE or chymase and to Angiotensin-(1–7) by neprilysin. We found that in primary cells the activity of ACE/chymase outcompeted neprilysin activity, which was reversed in the cell line, implicating a major importance of neprilysin in the cell line.

Neprilysin is a neutral endopeptidase that cleaves several peptides such as Endothelin-1 and -2, Substance P, Adrenomedullin, Bradykinin and mitogenic growth factors besides Angiotensin-I. The level of neprilysin activity plays an important role in malignancies and is downregulated in cancers (mainly by epigenetic mechanisms). neprilysin plays an important role in pulmonary hypertension and vascular remodeling in lung diseases [31].

The conversion of Angiotensin II to Angiotensin-(1–7) by ACE2 was similar in magnitude between the primary cells and the cell line. EVs converted Angiotensin I to Angiotensin II, primarily by ACE activity in primary cells and by chymase activity in the cell line. However, despite the well-defined cell culture environment in our assay, it is difficult to discern whether chymase activity is an integral component of EVs or whether the enzyme secreted by the cells gets loosely attached to EVs or is concentrated together with EVs by ultrafiltration. chymase forms Angiotensin II very efficiently, which is reflected in an increased Ang II formation from the Ang I substrate. This enzyme was originally believed to be primarily released from mast cells and plays a role in inflammation and tissue remodeling, exerts proteolytic activity on growth and differentiation factors and activates proteinase-activated receptors and angiotensin [32]. However, it is now also known to occur in fibroblasts and endothelial cells [33,34]. chymase has an important role in angiogenesis during tumor progression, MMP activation in atherosclerosis, TGF-ß activation and endothelin-1 formation [34]. The chymase content of EVs has recently been shown to be dependent on disease status in the context of hypertension [35]. In parallel, ACE2 activity was also higher in EVs from the cell line, possibly representing a counter-regulatory means.

## 4. Materials and Methods

### 4.1. Isolation of Primary HLMVECs and Cell Culture

Human lung microvascular endothelial cells (HLMVECs) were isolated from tissue fragments as left-overs from donor lungs during lung transplantations essentially as described by Mackay et al. [36] but with some modifications. Briefly, tissues without visible arterioles, bronchioles and venules were digested with 0.2% type II collagenase (CL-2, Worthington, Lakewood, NJ, USA) in RPMI and 0.1% bovine serum albumin at room temperature for 2 h with agitation. After incubation, the remaining larger tissue pieces were removed and the cell suspension filtered on a 100 μm cell strainer. The cells passing through were centrifuged at 250× *g* for 5 min and cultured on cell culture plates coated with 2% gelatin in EGM^TM^-2MV medium (LONZA, Basel, Switzerland) with bullet kit supplements. Once small clusters of growing endothelial cell masses were identified by their cobblestone appearance, a first selection and purification were performed with combined CD31 Dynal beads (Invitrogen, Waltham, MA, USA) and Ulex europeaeus agglutinin-1 (UEA-1)-coupled Dynabeads. The coupling of UEA-1 (Sigma Aldrich, St. Louis, MO, USA) to tosyl-activated Dynabeads M-450 (Dynal, Oslo, Norway) was performed as described by Jackson et al. [37]. After one week of culture, another purification step was performed before the enriched cell population (HLMVEC289) was transferred to the transfection procedure.

### 4.2. Generation of the HLMVEC/SVTERT289 Cell Line 

For the establishment of a continuously growing cell line, HLMVECs were co-transfected with a plasmid carrying the early region of Simian Virus 40 (large T/small t), including the SV40 promoter/enhancer sequences [38,39] and a plasmid carrying the coding region of the catalytic subunit of human telomerase (hTERT) and neomycin phosphotransferase (neo) as selection marker. Transfectants were selected by outgrowth of cells expressing the SV40 early region and that were resistant to 200 µg/mL Geneticin sulfate (G418, Invitrogen, San Diego, CA, USA). Cell clones were enriched and the resulting mass culture used for establishment of a master cell bank (HLMVEC/SVTERT289). The number of passages was calculated from the first split after the selection of gene transfer.

### 4.3. Basic Characterization of the Cell Line

#### 4.3.1. Flow Cytometry of Surface Labeled Cells

Cells were gently trypsinized and fixed for 15 min with 4% paraformaldehyde on a rotating wheel. After washing with PBS and centrifugation at 200× *g* for 5 min, the cell pellet was resuspended in 100 μL PBS and stained with the following antibodies for surface antigens: PerCP/Cy5.5 anti-human CD45; Pacific Blue anti-human CD54; Brilliant Violet 605 anti-human CD90; Alexa Fluor 700 anti-human CD31 (all from Biolegend, San Diego, CA, USA) and UEA-1 Fluorescein (Vector Labs, Newark, CA, USA).

#### 4.3.2. Immunofluorescence

Expression of SV40 large T was assessed by immunofluorescence. Cells were seeded in IBIDI slides (IBIDI, Gräfelfing, Germany) and fixed with 4% HistoFix (Carl Roth, Karlsruhe, Germany) for 10 min at room temperature. Blocking and permeabilization was performed by incubation for 20 min with 5% BSA/0.3% Triton-X-100. The primary antibody, mouse anti-SV40 T Antigen (Oncogene, Merck Millipore, Burlington, MA, USA), was diluted 1:100 in 5% BSA. The secondary antibody, anti-mouse AF488 (Jackson ImmunoResearch, West Grove, PA; USA), was diluted 1:1000 in 5% BSA. DNA counterstaining was performed using Vybrant^®^ DyeCycle™ Violet Stain (Invitrogen, Thermo Fisher Scientific, Waltham, MA, USA). Imaging was performed on an EVOS M5000 fluorescent microscope (Invitrogen-ThermoFisher Scientific, Waltham, MA, USA).

#### 4.3.3. TRAP Assay

Telomerase activity in HLMVECs and HLMVEC/SVTERT289 cells was determined using the TRAPeze^®^ RT Telomerase Detection Kit (Chemicon^®^, Merck Millipore, Burlington, MA, USA) according to manufacturer’s protocol. In brief, cultivated cells were detached and lysed in CHAPS buffer in a growing state at a concentration of 1000 cells/µL. For each sample, an aliquot was heat-inactivated for 10 min at 85 °C as a negative control. In addition to the samples, a TSR8 standard, a positive control (tumor cells provided in the kit, 1000 cells/µL), a negative control (CHAPS buffer) and a non-template control (H_2_O) were analyzed. An amount of 1 µL of standard/sample/control was analyzed in triplicate. Bio-Rad CFX Opus 96 was used for the quantification of the assay (Bio Rad Laboratories, Hercules, CA, USA).

#### 4.3.4. Tube Formation Assay

Capillary tube formation was assessed in primary HLMVECs as well as HLMVEC/SVTERT289 cells by a Matrigel assay. Matrigel (Corning, NY, USA) was thawed overnight on ice and evenly layered on each well of a pre-cooled 24-well cell culture plate. The plate was incubated at 37 °C for 30 min to allow the Matrigel to polymerize. Excess liquid was carefully removed without disturbing the matrix layer. HLMVEC/SVTERT289 cells were cultured to a desired confluency, trypsinized and resuspended in growth media with 10% FBS. A total of 12 × 10^4^ cells were carefully added to each well onto the Matrigel. Angiogenic stimulants, vascular endothelial growth factor (VEGF, Promocell, Heidelberg, Germany) and basic fibroblast growth factor (bFGF, Sigma, St. Louis, MO, USA) were added to the respective wells at a final concentration of 50 ng/mL, and the plate was incubated at 37 °C. Tube formation was monitored microscopically every few hours. In primary HLMVECs, tube formation peaked at 14–16 h, whereas in HLMVEC/SVTERT289 cells, peak tube formation was observed at 4 h. At the time of peak tube formation, the medium was gently aspirated without disrupting the tube network. Wells were washed twice with PBS. Calcein AM dye (Corning, NY, USA) was added to each well before incubation for 30–40 min at 37 °C. The Calcein solution was aspirated and the wells washed with PBS. The plate was immediately visualized under a fluorescent microscope (LSM 700, Zeiss, Oberkochen, Germany). Tube formation was quantified by measuring the number and length of tubes formed under each condition. Primary cells were used at passage 5 and HLMVEC/SVTERT289 cells were used at passage 11–12.

#### 4.3.5. Isolation of Extracellular Vesicles

Cell culture supernatants were centrifuged for 10 min at 200× *g* to remove debris and 10 min at 2000 g to remove apoptotic bodies and were then concentrated by ultrafiltration using 100 kDa filters (Amicon Ultra-15; Millipore, Burlington, MA, USA) at 3200 g with 3 washes to remove non-vesicle soluble proteins.

#### 4.3.6. Flow Cytometry of EVs

Quantitative assessment of EVs with surface-exposed phosphatidylserine, Tissue Factor (Factor III; TF), CD143 (angiotensin converting enzyme, ACE) and ACE2 was essentially performed as described in detail in [40].

Briefly, extracellular vesicles were identified by flow cytometry using Calcein and Lactadherin (phosphatidylserine-positive EVs; microvesicles) and were subtyped with regard to reactivity towards TF, ACE, and ACE2 antibodies. Staining antibodies (anti-human TF-PE (clone HTF-1; LSBio Lynnwood, WA, USA), anti-human ACE-PE/Cy7 (Biolegend, St. Diego, CA, USA, clone 5–369), anti-human ACE2-AF750 (R&D Systems, Minneapolis, MI, USA; clone 535919)) were centrifuged for 10 min at 17,000× *g*. A total of 6.5 μL of previously titrated antibody mixture was added to 25 μL PBS and 10 μL concentrated vesicle sample and incubated for 60 min on ice. Finally, 10 μL bovine Lactadherin-AF647 (1:10) (CellSystems, Troisdorf, Germany) and 50 μL Calcein-AM (Biolegend, St.Diego, CA, USA) was added and the incubation continued for an additional 30 min. Samples were fixed with 50 μL 4% paraformaldehyde and diluted with 850 μL PBS before being analyzed on a CytoFlex LX flow cytometer (Beckman Coulter, Brea, CA, USA) equipped with 5 lasers (355, 405, 488, 561, 638 nm; scatter signal at 405 nm laser). As additional controls, unstained samples were prepared in which staining antibodies were replaced with the equivalent amount of PBS. Staining antibodies were run in PBS alone to account for potential background signals. Data were obtained from the Cytoflex using CytExpert software (V2.4; Beckman Coulter Life Sciences, Krefeld, Germany).

#### 4.3.7. Proteomic Analysis of EVs

##### Sample Preparation

The protein content of the isolated exosome samples was digested with trypsin using S-Trap micro spin columns (ProtiFi, Fairport, NY, USA) according to the vendor’s protocol. Briefly, samples were mixed with 6 μL of 20% SDS, sonicated for 5 min, dried in a speed-vac concentrator and resuspended in 23 µL of 50 mM triethylammonium bicarbonate (TEAB pH = 8.5). Proteins were reduced with 5 mM tris(2-carboxyethyl)phosphine (TCEP) at 55 °C for 15 min and alkylated with 20 mM methyl-methanethiosulfonate (MMTS) at room temperature for 10 min. The pH was decreased below 1 with the addition of phosphoric acid and proteins were trapped on the S-Trap™ micro spin column. After washing, 1 µg of trypsin (Trypsin Protease, MS grade; Pierce, Appleton, WI, USA) was added and the mixture incubated at 47 °C for 2 h. Tryptic peptides were eluted and dried before TMT labeling. The resulting peptide mixtures were labeled with Tandem Mass Tag System (TMT) 10 plex isobaric label reagents (Thermo Scientific, Waltham, MA, USA) according to the standard labeling protocol. Briefly, dried sample digests were dissolved in 50 µL of 100 mM TEAB buffer and 20 µL of TMT reagent (0.8 mg resuspended in 41 µL anhydrous acetonitrile) was added and incubated at room temperature for 1 h. The excess reagent was quenched by the addition of hydroxylamine. Equal amounts of each sample (corresponding to the same donor) were combined and dried down before fractionation.

The High pH Reversed-Phase Peptide Fractionation Kit (Pierce, Appleton, WI, USA) was used to pre-fractionate the pooled TMT labelled samples before LC-MS/MS analysis according to the vendor’s protocol. Briefly, the sample was loaded onto the column in 0.1% trifluoroacetic acid (TFA) and eluted successively with 5%, 10%, 12.5%, 15%, 17.5%, 20%, 22.5%, 25% and 50% acetonitrile with 0.1% Triethylamine. Eluates were dried down, redissolved in 1% TFA and loaded on Evotips (Evosep Biosystems, Odense, Denmark) according to the vendor’s protocol.

##### LC-MS/MS Analysis

Samples were analyzed by LC-MS/MS using an Evosep One system (Evosep Biosystems, Odense, Denmark) on-line coupled to an Orbitrap Fusion Lumos Tribrid (Thermo Scientific, Waltham, MA, USA) mass spectrometer equipped with FAIMS interface. The 15-samples-per-day method with its preprogrammed gradient was applied using an endurance column (EV-1137 column, 15 cm × 150 µm, 1.5 µm); the flow rate was 220 nl/min. Peptides eluted from the column were analyzed using −45, −60 and −75 FAIMS compensation voltages in 3 s cycles, selecting the most abundant multiply charged ions (z = 2–6, m/z range:400–1400) for HCD fragmentation (normalized collision energy: 38%) following each MS1 scan. Both MS and MS/MS spectra were collected in the Orbitrap analyzer with resolutions of 120,000 or 15,000, respectively.

##### Data Analysis and Statistics

Database search and quantitative analysis were conducted using Proteome Discoverer (v3.0 SP1, Thermo Scientific, Waltham, MA, USA) software. Proteins were identified using Sequest HT software (ThermoFisher Scientific, Waltham, MA, USA) with the following parameters: database: SwissProt Homo sapiens sequences (2022.12.14. version, 20,330 sequences) concatenated with a reversed version for each entry; enzyme: trypsin allowing maximum two missed cleavage sites; modifications: static: methylthio on Cys, TMT label on any N terminus and on Lysine residues; dynamic: oxidation of Met, deamidation of Gln or Asn, allowing up to 4 variable modifications/peptide; mass accuracy: 5 ppm and 0.02 Da for precursor and fragment ions, respectively. The peptide-level false discovery rate was below 1% for all samples, as estimated by the incidence of reversed sequence identifications.

For quantification, the S/N (signal to noise) values of the reporter ions were used. Normalization was performed on the total peptide amount per channel. Protein ratio calculations were based on pairwise ratios and a background-based *t*-test was used for hypothesis testing.

### 4.4. In Vitro Model of Mechanical Stress Exposure

#### Cell Stretching-Flexcell^®^ System

Cells were seeded into 6-well FlexCell^®^-plates (200,000 per well) with a Collagen 1 coating (BioFlex plates Collagen I; FlexCell^®^ international, Hillsborough, NC, USA) using a seeding regime of 24 h. Cell stretching was performed using a FlexCell^®^ FX5KTM Tension System (FlexCell^®^ International, Hillsborough, NC, USA) (including FlexLink FX5000T-FLK, Trivac^®^ B/D8 B vacuum pump and FX-5000 V1.0 Software).

Prior to starting the elongation program, the full attachment medium was replaced with 2 mL serum-free medium. The high elongation regime included 14% (max. 54.71 kPa pressure) or 21% (max. 84.78 kPa pressure) elongation. Stretching followed a sinus wave with a frequency of 0.25 Hz. After completion of the regime, the supernatant was analyzed by flow cytometry, ELISA, RAS enzyme activity assays and quantitative proteomics, respectively, and cells were analyzed by RAS enzyme activity assays and quantitative real-time PCR.

### 4.5. Other Analytical Methods

#### 4.5.1. Western Blot Analysis

Protein expression of the ACE protein was analyzed from HLMVEC/SVTERT289 cells (unstretched and stretched for 24, 48 h and 72 h) lysed in RIPA buffer by SDS-PAGE (8% gel) and Western blotting using the primary antibodies rabbit anti-ACE/CD143 (JM59-32; NOVUS Biologicals, Littleton, CO, USA) and mouse anti-GAPDH loading control antibody (MA5-15738; Invitrogen, Waltham, MA, USA).

#### 4.5.2. Quantitative Real-Time Polymerase Chain Reaction

Cellular mRNA was isolated using the RNAeasy mini Kit (Qiagen, Hilden, Germany) and 500 ng of this reverse transcribed (Perfecta qScript; Quanta Biosciences, Gaithersburg, MD, USA). Quantitative real-time PCR was performed using Perfecta SYBRgreen fast Mix (Quanta Biosciences, Gaithersburg, MD, USA) on a RotorGene Q (Qiagen, Hilden, Germany) with the following temperature program: 3 min of 95 °C and 40 cycles, 30 s of 95 °C, 15 s of 60 °C and 10 s 72 °C.

The following primers were used: (Table 1).

#### 4.5.3. Activity Analysis of RAS Enzymes

The cell surface and vesicle-bound activity of the RAS components ACE, ACE2, neprilysin and chymase was analyzed by Attoquant Diagnostics using liquid chromatography–mass spectrometry/mass spectroscopy (LC-MS/MS). As a principle of the assays, Angiotensin metabolism of the enzymes ACE (Ang I => Ang II conversion), chymase (Ang I => Ang II conversion), neprilysin (Ang I => Ang 1–7 conversion) and ACE2 (Ang II => Ang 1–7 conversion) was determined in the presence and absence of their specific inhibitor.

Cells were grown in 6-well plates to sub-confluence. Cell stretch was performed in serum-free medium for each plate (0%, 14% and 21% stretch) for the indicated duration (48 h or 72 h). Supernatants were pooled, cleared by low-speed centrifugation (10 min 200× *g* and 10 min 2000× *g*), concentrated by ultracentrifugation on an Amicon Ultra-15 filter (cut-off 30 kDa) and stored at −20 °C for the analysis of vesicles. Cells after stretching were washed with serum-free medium and incubated with 800 μL of reaction mixes (PBS, 1 mM ZnCl_2_, pH 7.4, including the specific substrates as spike-in: Angiotensin I for ACE, chymase and neprilysin and Angiotensin II for ACE2) without or with specific enzyme inhibitors (ACE inhibitor: 10 µM Lisinopril; ACE2 Inhibitor: 10 µM MLN-4760; chymase inhibitor: 10 µM Chymostatin; neprilysin inhibitor: 10 µM LBQ657) or no inhibitors (control) for 60 min at 37 °C. For quality control purposes, the reaction mixes were additionally incubated with the following recombinant proteins: 1 ng/mL ACE2, 10 ng/mL ACE, 10 ng/mL neprilysin or 1 ng/mL chymase as positive controls for analyses. Supernatants were diluted in equal reaction mixes. Following incubation, the reaction mixes with cells or supernatants were acidified (final conc: 1% formic acid in 10% acetonitrile) and stored at −20 °C for LC-MS/MS analysis. Acidified samples were thawed on ice and spiked with stable isotope labeled internal standards for each angiotensin metabolite at a concentration of 200 pg/mL. The samples subsequently underwent C-18-based solid-phase extraction and were subjected to LC-MS/MS analysis using a reversed-phase analytical column operating in line with a Xevo TQ-S triple quadruple mass spectrometer (Waters, Milford, MA, USA). Internal standards were used to correct for the peptide recovery of the sample preparation procedure for each analyte in each individual sample.

The product formation (Ang II formation for ACE and chymase; Ang 1–7 formation for ACE2 and neprilysin) measured in wells with the specific inhibitor of the enzyme were subtracted from controls (without inhibitor) to determine the specific enzyme activities.

For technical reasons, data were obtained from 2 or 3 different experiments (depending on the analyte), each performed with at least technical duplicates.

## 5. Conclusions

Our novel lung microvascular endothelial cell line, HLMVEC/SVTERT289 (immortalized by using hTERT and SV40 T-antigen), has proven to proliferate like common immortalized cell lines and at the same time maintains to a large extent the morphology and basic characteristics of the primary cells that they are derived from. They exhibit a similar response to cyclic stretch with regard to gene expression and the release of extracellular vesicles, which implicates their putative suitability as model for conditions like ventilator-induced lung injury and maybe also other lung diseases. Significant differences were observed with regard to the enzymatic activities of components of the renin–angiotensin system. In the cell line, it is noticed that neprilysin displays a greater ability to form Ang1–7 from Ang I and the extracellular vesicles released from the cell line mainly rely on chymase instead of ACE activity to form Ang II from Ang I. These cells may be a useful initial model system to study the molecular mechanisms in the pulmonary microvasculature, especially because the availability of analogous primary human cells is very limited.

## Figures and Tables

**Table 1:** Primer sequences for qRT-PCR.

Gene	Forward Primer	Reverse Primer	Tm (°C)
ß-Actin	CATGTACGTTGCTATCCAGGC	CTCCTTAATGTCACGCACGAT	59.8/57.9
ACE	GGAGGAATATGACCGGACATCC	TGGTTGGCTATTTGCATGTTCTT	62.1/57.1
neprilysin	AGAAGAAACAGCGGATGGACTCC	CATAGAGTGCGATCATTGTCACA	60.3/58.9
Interleukin-6	ACTCACCTCTTCAGAACGAATTG	CCATCTTTGGAAGGTTCAGGTTG	58.9/60.6
VEGFA	AGGGCAGAATCATCACGAAGT	AGGGTCTCGATTGGATGGCA	57.9/59.4
VE-PTP	GGGCTCACCCCTGTAACTTTAGC	TCTATCCGAAAGGTAGGGCAC	62.1/59.8
PECAM1	AACAGTGTTGACATGAAGAGCC	TGTAAAACAGCAGTCATCCTT	58.4/56.5
ICAM1	ATGCCCAGACATCTGTGTCC	GGGGTCTCTATGCCCAACAA	59.4/59.4
ZO1/TJP1	CAACATACAGTGACGCTTCACA	CACTATTGACGTTTCCCCACTC	58.4/60.3
OCLN	ACAAGCGGTTTTATCCAGAGTC	GTCATCCACAGGCGAAGTTAAT	58.4/58.4
RAGE	GTGTCCTTCCCAACGGCTC	ATTGCCTGGCACCGGAAAA	61.0/56.7
Angpt2	AACTTTCGGAAGAGCATGGAC	CGAGTCATCGTATTCGAGCGG.	57.9/61.8

## Data Availability

All individual data from experiments presented in the manuscript are available upon request from the corresponding author.

## Supplementary Materials

The following supporting information can be downloaded at: https://www.mdpi.com/article/10.3390/ijms26020683/s1.
